# Using entropy-driven amplifier circuit response to build nonlinear model under the influence of Lévy jump

**DOI:** 10.1186/s12859-021-04331-0

**Published:** 2022-01-20

**Authors:** Hao Fu, Hui Lv, Qiang Zhang

**Affiliations:** 1 Key Laboratory of Advanced Design and Intelligent Computing, Ministry of Education, School of Software Engineering, Dalian University, Dalian, 116622 China; 2 State Key Laboratory of Synthetical Automation for Process Industries, Northeastern University, Shenyang, 110004 China; 3 School of Computer Science and Technology, Dalian University of Technology, Dalian, 116024 China

**Keywords:** Entropy-driven amplifier, Nonlinear system model, Lévy jump, Lyapunov function

## Abstract

**Background:**

Bioinformatics is a subject produced by the combination of life science and computer science. It mainly uses computer technology to study the laws of biological systems. The design and realization of DNA circuit reaction is one of the important contents of bioinformatics.

**Results:**

In this paper, nonlinear dynamic system model with Lévy jump based on entropy-driven amplifier (EDA) circuit response is studied. Firstly, nonlinear biochemical reaction system model is established based on EDA circuit response. Considering the influence of disturbance factors on the system, nonlinear biochemical reaction system with Lévy jump is built. Secondly, in order to prove that the constructed system conforms to the actual meaning, the existence and uniqueness of the system solution is analyzed. Next, the sufficient conditions for the end and continuation of EDA circuit reaction are certified. Finally, the correctness of the theoretical results is proved by numerical simulation, and the reactivity of THTSignal in EDA circuit under different noise intensity is verified.

**Conclusions:**

In EDA circuit reaction, the intensity of external noise has a significant impact on the system. The end of EDA circuit reaction is closely related to the intensity of Lévy noise, and Lévy jump has a significant impact on the nature of biochemical reaction system.

## Background

Bioinformatics is a discipline produced by the combination of life science and computer science, which mainly uses computer technology to study the laws of biological systems. DNA circuit reaction is one of the important research objects of bioinformatics, which is applied to biological systems [1] and other fields. Researchers have designed and provided research subjects for various types of DNA circuits, and the study of DNA circuit reactions can facilitate the development of information biology.

DNA circuits play a key role in signal amplification and information regulation of biomolecular engineering systems. In recent years, more and more advanced and more complex synthesis circuits have been designed to build more and more reliable, efficient and complex molecular signal pathways. The purpose of many bioinformatics studies is to identify markers or characteristics, which can be used to distinguish different groups [2, 3]. Fluorescence labeling is often used in DNA circuit reaction [4], and fluorescence is more expensive. Using ThT instead of fluorescence as a reporter can save cost and make it easier to achieve. Synthetic DNA circuits transmit complex information through two main catalytic mechanisms: enzyme-dependent DNA cascades [5, 6] and entropy-driven DNA catalytic reactions [7–10]. Especially entropy-driven circuit is attractive due to its catalytic ability, signal amplification and programmable network [11]. Zhang et al. [12] first propose entropy-driven circuit, which provides a simple, fast, modular, combinable and robust amplifying circuit element. Entropy-driven DNA circuits have been widely used in logic operations [13, 14], nanostructure formation [11], DNA computing[15, 16], molecular detection [17] and molecular engineering [18]. Zhang et al. [19] implement a cutting-assisted recovery strategy for reactants in an entropy-driven DNA circuit. In [20], a molecular engineering, entropy-driven 3D DNA amplifier is developed, which can work in response to specific intracellular mRNA targets in living cells. In [21], a DNA amplifier functionalized MOF particle is developed, which can be used to detect and image intracellular mRNAs. Damase et al. [22] design an EDA circuit mechanism with Thioflavin T detection. Therefore, entropy-driven circuit plays an important role in bioinformation system.

In nature, most systems are nonlinear. Therefore, in order to better analyze nonlinear systems, it is necessary to establish mathematical models of nonlinear systems. Many nonlinear system modeling methods [23–28] have been proposed. Based on the above methods, nonlinear system models can be established [29, 30], and sensitivity analysis [31, 32], stability analysis [30, 33, 34] and bifurcation analysis [35–37] can be performed on them. A parametric model of dosetime response is proposed in [38], demonstrating the effectiveness of our model for all available anticancer compounds. In real life, it is full of randomness, and random disturbance is inevitable. Many scholars have studied the infectious disease system and population system affected by Lévy noise. In biochemical reaction, it is often subjected to sudden and severe disturbances, such as pressure shock, thermal shock, and sudden addition of catalyst, etc. These factors will cause the reaction to jump. Lévy jump is often used in infectious disease models [39–42], proving that the influence of Lévy noise can lead to the extinction of diseases. Lévy jumps are also used in epidemic models [43] and virus dynamics models [44]. [45] and [46] introduce the introduction of Lévy jump in the predator system, and analyze the sufficient conditions for the species’ continued survival and extinction. Lu et al. [47] introduce Lévy jump in the Lotka-Volterra competition model and analyze the conditions of system stability. In [48], Lévy jump is added to the symbiosis model and analyzed the sufficient conditions for the stability of the system distribution. Gao et al. [49] add Lévy jump to the multi-molecule biochemical reaction model, and prove the conditions for the end and duration of the system reaction. Gaussian white noise is only the idealization of all kinds of random noise in reality. It can only describe the small disturbance near the mean value, but cannot simulate the large-scale random disturbance, while Lévy noise can describe the large-scale random disturbance. In biochemical reaction system, there are few studies with Lévy noise. Therefore, adding disturbance such as Lévy jump into the system can better understand the properties of the system.

In biological information system, external noise often has a greater impact on the system. Since the temperature will change during the reaction of EDA circuit, resulting in a large-scale random disturbance, this phenomenon needs to be described by a stochastic differential equation driven by Lévy jump. Focusing on the above-mentioned problems, this paper discusses nonlinear biochemical reaction system model with Lévy jump based on EDA circuit reaction. For the first time, Lévy jump is introduced into DNA strand replacement system to study the properties of the system. First, nonlinear biochemical reaction system model based on EDA circuit reaction is established according to the law of conservation of mass and mathematical modeling. Considering the influence of random disturbance on EDA circuit reaction system, a nonlinear biochemical reaction system model with Lévy jumps is established. Then, considering the influence of Lévy jump on the response of EDA circuit, the sufficient conditions for the end and the continuation of EDA response are analyzed. When noise intensity is large enough or meets the appropriate conditions, Lévy jump will force EDA circuit reaction to end. At this time, the concentration of reactants in EDA circuit reaction decreases to the lowest, the reaction activity of ThTSignal reaches the maximum, and the fluorescence intensity of EDA circuit reaction reaches the maximum. When noise intensity is small enough, Lévy jump makes the reaction continue. At this point, the concentration of reactants in EDA circuit reaction reaches dynamic equilibrium and is not completely consumed. The low reaction activity of ThTSignal leads to the decrease of fluorescence intensity of EDA circuit reaction. Finally, the accuracy of the theoretical results is verified by numerical simulation.

The main contributions of this research are as follows:

1. For the first time, Lévy jymp is introduced into DNA strand displacement reaction of EDA circuit reaction, and the stochastic differential equation system model driven by Lévy jump is analyzed.
2. The nonlinear biochemical reaction system with Lévy jump is established, which transforms EDA circuit reaction process into nonlinear mathematical model, and provides a theoretical basis for further research on EDA circuit reaction.
3. The sufficient conditions for the end and continuation of EDA circuit reaction under the influence of Lévy jump are analyzed, and the influence of noise intensity on ThTSignal reaction activity in EDA circuit reaction is studied.

The rest of this article is organized as follows: in section two, based on EDA reaction, nonlinear biochemical reaction model is established, and the disturbance factor is considered, Lévy jump is introduced into the system, and nonlinear biochemical reaction system model with Lévy jump is built. In section three, the existence and uniqueness of the positive solution of the system is analyzed. In section four, the sufficient conditions for the end and continuation of EDA circuit reaction are certified, and the reactivity of ThTSignal under different noise intensity is attested. In section five, the above conclusions are verified by numerical simulation.

## Methods

In this part, the modeling method of stochastic differential equations with Lévy jumps will be introduced. In EDA circuit reaction, it often suffers from sudden and severe disturbances, such as pressure shock, thermal shock, and sudden addition of catalyst. These factors will cause a jump in the response of EDA circuit. However, Gaussian white noise can only describe small disturbances, but cannot simulate large random disturbances. Therefore, in order to describe this type of noise, it is reasonable and necessary to introduce Lévy jump process in EDA circuit reaction model and use the stochastic differential equation driven by jump process to explain these phenomena in EDA circuit reaction.

### Modeling of nonlinear biochemical reaction system based on EDA reaction

**Fig. 1:**
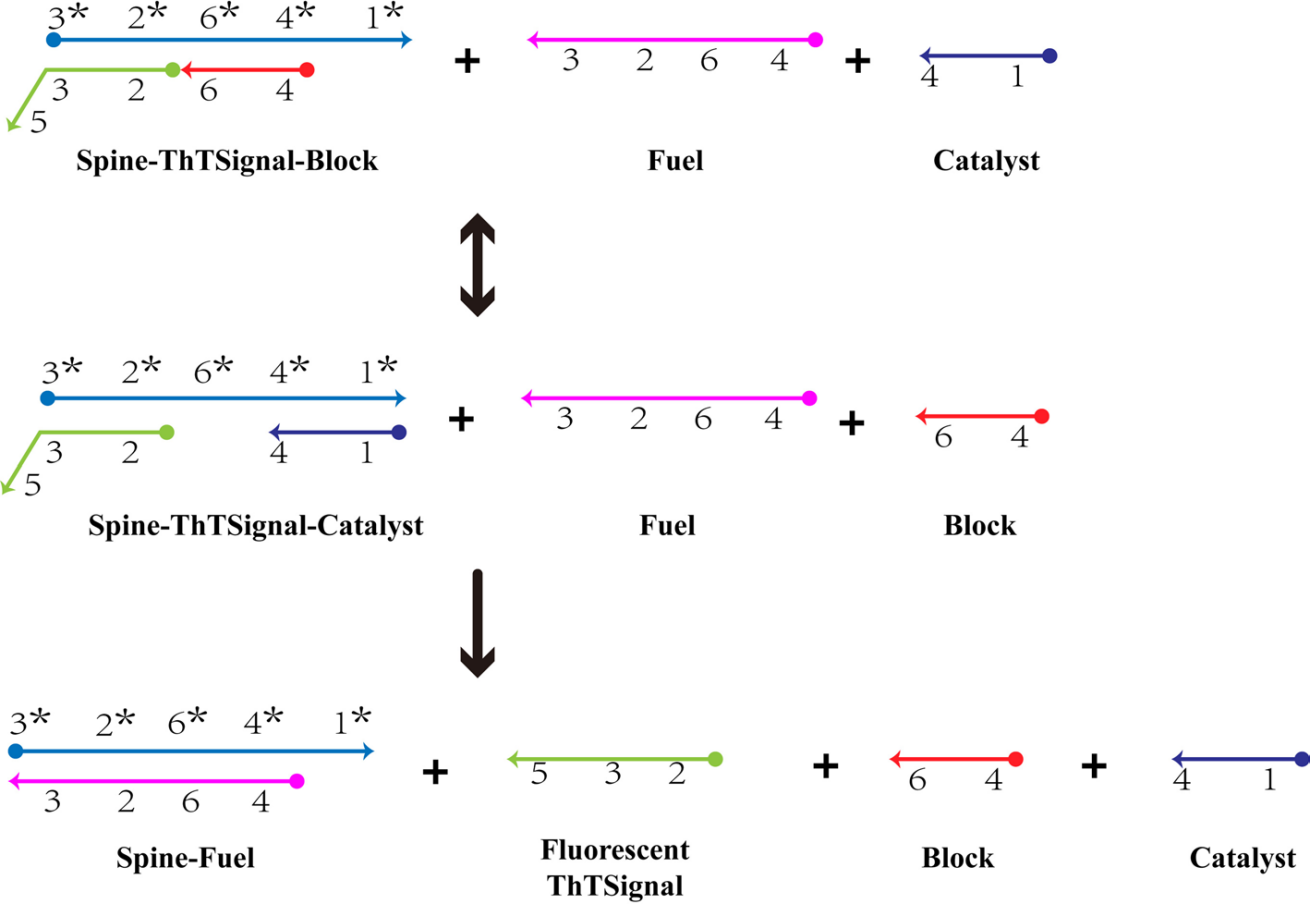
EDA circuit reaction diagram

In this section, a mathematical model of nonlinear biochemical reaction system based on EDA circuit reaction [22] will be established. Among them, the schematic diagram of EDA circuit response is shown in Fig. 1.

In EDA circuit reaction, ThTSignal is used as the unlabeled reporter of DNA-DNA reaction activity by using the fluorescence characteristic of ThT binding with DNA sequence. In order to further study the activity of ThTSignal reaction, we establish nonlinear biochemical reaction system model based on EDA circuit reaction. The reaction equation of EDA circuit is as follows

1 $$\begin{aligned} \left\{ \begin{aligned}&{\mathrm{Spine-ThTSignal-Block}}+{\mathrm{Catalyst}}\underset{{{k}_{-1}}}{\overset{k{}_{1}}{\mathop {\rightleftharpoons }}}\,{\mathrm{Spine-ThTSignal-Catalyst}}+{\mathrm{Block}} \\& {\mathrm{Spine-ThTSignal-Catalyst}}+{\mathrm{Fuel}}\overset{{{k}_{2}}}{\mathop {\rightarrow }}\,{\mathrm{Spine-Fuel}}+{\mathrm{ThTSignal}}+{\mathrm{Catalyst}}, \end{aligned} \right. \end{aligned}$$

where ${{k}_{1}}$, ${{k}_{-1}}$ and ${{k}_{2}}$ represent the reaction rate. For convenience, the reactants Spine-ThTSignal-Block, Catalyst, the intermediate product Spine-ThTSignal-Catalyst, waste Block, Fuel, the product Spine-Fuel, and the fluorescent product ThTSignal are respectively used as *x*, *a*, *y*, *b*, *c*, *d* and *e*. Then the net reaction equation of EDA circuit reaction can be obtained as

2 $$\begin{aligned} \left\{ \begin{aligned}&x+a\underset{{{k}_{-1}}}{\overset{{{k}_{1}}}{\mathop {\rightleftharpoons }}}\,y+b \\&y+c\overset{{{k}_{2}}}{\mathop {\rightarrow }}\,d+e+a, \\ \end{aligned} \right. \end{aligned}$$

According to the net reaction equation of EDA circuit reaction, nonlinear mathematical model of EDA circuit response can be established by using mathematical modeling method.

3 $$\begin{aligned} \left\{ \begin{aligned}&\dot{x}\left( t \right) ={{k}_{-1}}b\left( t \right) y\left( t \right) -{{k}_{1}}a\left( t \right) x\left( t \right) \\&\dot{a}\left( t \right) ={{k}_{-1}}b\left( t \right) y\left( t \right) -{{k}_{1}}a\left( t \right) x\left( t \right) +{{k}_{2}}c\left( t \right) y\left( t \right) \\&\dot{y}\left( t \right) ={{k}_{1}}a\left( t \right) x\left( t \right) -{{k}_{-1}}b\left( t \right) y\left( t \right) -{{k}_{2}}c\left( t \right) y\left( t \right) \\&\dot{b}\left( t \right) ={{k}_{1}}a\left( t \right) x\left( t \right) -{{k}_{-1}}b\left( t \right) y\left( t \right) \\&\dot{c}\left( t \right) =-{{k}_{2}}c\left( t \right) y\left( t \right) \\&\dot{d}\left( t \right) ={{k}_{2}}c\left( t \right) y\left( t \right) \\&\dot{e}\left( t \right) ={{k}_{2}}c\left( t \right) y\left( t \right) , \\ \end{aligned} \right. \end{aligned}$$

where $x\left( t \right)$, $a\left( t \right)$, $y\left( t \right)$, $b\left( t \right)$, $c\left( t \right)$, $d\left( t \right)$ and $e\left( t \right)$ respectively represent the concentration of reactant *x*, catalyst *a*, intermediate product *y*, waste *b*, fuel *c*, product *d* and fluorescent product *e* at time *t*.

Based on the net reaction equation (2) of EDA circuit reaction, according to the law of conservation of mass, the relationship between the initial concentration of the reactant and the concentration of the product can be obtained as

4 $$\begin{aligned} \left\{ \begin{aligned}&{{x}_{0}}=x\left( t \right) +y\left( t \right) +e\left( t \right) =x\left( t \right) +b\left( t \right) \\&{{a}_{0}}=a\left( t \right) +y\left( t \right) \\&{{c}_{0}}=c\left( t \right) +e\left( t \right) , \\ \end{aligned} \right. \end{aligned}$$

where ${{x}_{0}}$, ${{a}_{0}}$ and ${{c}_{0}}$ respectively represent the initial concentration of reactants *x*, *a* and *c*.

On the basis of the mathematical model of nonlinear biochemical reaction system based on EDA circuit reaction (3) and the initial concentration relationship of the reactants of EDA reaction (4), the following nonlinear biochemical reaction system model can be obtained

5 $$\begin{aligned} \left\{ \begin{aligned}&\frac{dx}{dt}=-{{a}_{1}}x\left( t \right) +{{b}_{1}}y\left( t \right) +{{c}_{1}}x\left( t \right) y\left( t \right) \\&\frac{dy}{dt}={{a}_{1}}x\left( t \right) -{{b}_{2}}y\left( t \right) +{{c}_{2}}x\left( t \right) y\left( t \right) -{{k}_{2}}{{y}^{2}}\left( t \right) , \\ \end{aligned} \right. \end{aligned}$$

where $${{a}_{1}}={{k}_{1}}{{a}_{0}}$$, $${{b}_{1}}={{k}_{-1}}{{x}_{0}}$$, $${{c}_{1}}={{k}_{-1}}-{{k}_{1}}$$, $${{b}_{2}}={{k}_{-1}}{{x}_{0}}+{{k}_{2}}\left( {{c}_{0}}-{{x}_{0}} \right)$$, $${{c}_{2}}={{k}_{-1}}-{{k}_{1}}-{{k}_{2}}$$.

#### Remark 1

In view of the complex calculation process and large amount of calculation when analyzing system characteristics, the dimensionality reduction of the system according to the law of conservation of mass can not only simplify EDA circuit reaction system, but also reduce the difficulty of calculation.

#### Remark 2

The use of mathematical modeling methods to model EDA circuit reaction system can more intuitively describe biological information system and make the abstract problems concrete, so that mathematical methods can be used to solve biological information system problems.

### Modeling of nonlinear biochemical reaction system with Lévy jumps based on EDA reaction

In real life, EDA circuit reactions may suffer sudden and severe disturbances, such as sudden addition of catalysts, changes in temperature, and so on. These factors will cause EDA circuit reactions to produce large random disturbances, and the resulting phenomenon cannot be accurately described by model (5). Therefore, Lévy jump is added to EDA circuit response model, so that the model can more accurately describe the reaction activity of ThTSignal in EDA circuit response. The following is nonlinear biochemical reaction model with Lévy jump based on EDA circuit reaction.

6 $$\begin{aligned} \left\{ \begin{aligned} dx =&\left( -{{a}_{1}}x\left( {{t}^{-}} \right) +{{b}_{1}}y\left( {{t}^{-}} \right) +{{c}_{1}}x\left( {{t}^{-}} \right) y\left( {{t}^{-}} \right) \right) dt\\&-x\left( {{t}^{-}} \right) y\left( {{t}^{-}} \right) \left( \sigma dB\left( t \right) +\int _{\mathbb {Y}}{\gamma \left( u \right) \tilde{N}\left( dt,du \right) } \right) \\ dy =&\left( {{a}_{1}}x\left( {{t}^{-}} \right) -{{b}_{2}}y\left( {{t}^{-}} \right) +{{c}_{2}}x\left( {{t}^{-}} \right) y\left( {{t}^{-}} \right) -{{k}_{2}}{{y}^{2}}\left( {{t}^{-}} \right) \right) dt\\&+x\left( {{t}^{-}} \right) y\left( {{t}^{-}} \right) \left( \sigma dB\left( t \right) +\int _{\mathbb {Y}}{\gamma \left( u \right) \tilde{N}\left( dt,du \right) } \right) , \\ \end{aligned} \right. \end{aligned}$$

where $x\left( {{t}^{-}} \right)$ and $y\left( {{t}^{-}} \right)$ are the left limits of $x\left( t \right)$ and $y\left( t \right)$ respectively, $B\left( t \right)$ is the standard one-dimensional Brownian motion, $B\left( 0 \right) =0$, ${{\sigma }^{2}}>0$ are the intensity of white noise, $\tilde{N}$ is the compensated random measure defined by $\tilde{N}\left( dt,du \right) =N\left( dt,du \right) -\lambda \left( du \right) dt$, N is the Poisson counting measure, and $\lambda$ is the characteristic measure of N, which is defined on the finite measurable subset $\mathbb {Y}$ of $\left( 0,+\infty \right)$, $\lambda \left( \mathbb {Y} \right) <\infty$, $\gamma \left( u \right) :\mathbb {Y}\times \Omega \rightarrow \mathbb {R}$ are bounded continuous functions, and $\left| \gamma \left( u \right) \right| <l$, $l>0$ are constants. Assume that B and N are independent.

#### Remark 3

Using mathematical model with Lévy jumps to describe the response of EDA circuit can more realistically show the situation of biological information system in the real environment, thereby reducing the deviation and being closer to the real situation.

#### Assumption 1

$$\left| \left( \frac{{{k}_{2}}\left( {{x}_{0}}-{{c}_{0}} \right) }{2{{k}_{-1}}-{{k}_{2}}} \right) \gamma \left( u \right) \right| \le \delta <1\;{\mathrm{for}}\;{\mathrm{any}}\;u\in \mathbb {Y}.$$

Assuming that $\left( \Omega ,{\mathbf {F}},{{\left\{ {{\mathbf {F}}_{t}}\right\} }_{t\ge 0}},\mathbb {P} \right)$ be a complete probability space and it’s filtration ${{\left\{ {{\mathbf {F}}_{t}}\right\} }_{t\ge 0}}$ satisfies the general conditions (i.e. it’s right continuous and ${{\mathbf {F}}_{0}}$ contains all $\mathbb {P}$-null sets).

Define

$$\begin{aligned} {\mathbb{R}}_{{+}}^{d}=\left\{ x\in {{\mathbb {R}}^{d}}:{{x}_{i}}>0\; {\mathrm{for}}\;{\mathrm{all}}\;1\le i\le d \right\} ,\\ \bar{\mathbb {R}}_{{+}}^{d}=\left\{ x\in {{\mathbb {R}}^{d}}:{{x}_{i}}\ge 0\; {\mathrm{for}}\;{\mathrm{all}}\;1\le i\le d \right\} . \end{aligned}$$

In addition, $\left\langle f\left( t \right) \right\rangle$ is the mean value of the function $f\left( t \right)$ on $\left[ 0,\ \infty \right)$, that is $\left\langle f\left( t \right) \right\rangle =\frac{1}{t}\int _{0}^{t}{f\left( s \right) ds}$.

## Existence and uniqueness of positive solutions

In this section, it is proved that system (6) has a unique global positive solution for any initial value.

### Theorem 1

Let Assumption 1 holds, then for any given initial value $\left( x\left( 0 \right) ,y\left( 0 \right) \right) \in \mathbb {R}_{+}^{2}$, the model has a unique solution $\left( x\left( t \right) ,y\left( t \right) \right)$ at $t\ge 0$, and the solution will remain in $\mathbb {R}_{+}^{2}$ with probability 1, that is, $\left( x\left( t \right) ,y\left( t \right) \right) \in \mathbb {R}_{+}^{2}$ is almost surely (*a*.*s*.) for all $t\ge 0$.

### Proof

Since the coefficients of model (6) satisfy the local Lipschitz condition, for any given initial value $\left( x\left( 0 \right) ,y\left( 0 \right) \right) \in \mathbb {R}_{+}^{2}$, there exists a unique local solution $\left( x\left( t \right) ,y\left( t \right) \right)$ on $t\in \left[ 0,\ {{\tau }_{e}} \right)$, where ${{\tau }_{e}}$ is the explosion time. To prove that the solution is global, we only need to prove ${{\tau }_{e}}=\infty \;a.s.$ Therefore, let ${{m}_{0}}\ge 1$ be large enough that $\left( x\left( t \right) ,y\left( t \right) \right)$ is in the interval $\left[ \frac{1}{{{m}_{0}}},{{m}_{0}} \right]$. For each integer of $m\ge {{m}_{0}}$, the stop time is defined as follows

7 $$\begin{aligned} {{\tau }_{m}}=\inf \left\{ t\in \left[ 0,{{\tau }_{e}} \right] :\min \left\{ x\left( t \right) ,y\left( t \right) \right\} \le \frac{1}{m}or\max \left\{ x\left( t \right) ,y\left( t \right) \right\} \ge m \right\} , \end{aligned}$$

where assuming that $\inf \phi =\infty$ ($\phi$ be an empty set). Obviously, $\tau$ increases with $m\rightarrow \infty$. Let ${{\tau }_{\infty }}=\underset{m\rightarrow +\infty }{\mathop {\lim }}\,{{\tau }_{m}}$, thus ${{\tau }_{\infty }}\le {{\tau }_{e}}\;a.s.$ if ${{\tau }_{\infty }}=\infty \;a.s.$ is true, then ${{\tau }_{e}}=\infty \;a.s.$ and for any $t\ge 0$, there is $\left( x\left( t \right) ,y\left( t \right) \right) \in \mathbb {R}_{2}^{+}\;{\mathrm{a.s.}}$ In other words, in order to prove the conclusion, we only need to explain ${{\tau }_{\infty }}=\infty \;{\mathrm{a.s.}}$ On the contrary, there is a pair of constants $T>0$ and $\varepsilon \in \left( 0,1 \right)$ such that $P\left\{ {{t}_{\infty }}\le T \right\} >\varepsilon$, therefore, there is an integer ${{m}_{1}}\ge {{m}_{0}}$, such that

8 $$\begin{aligned} P\left\{ {{t}_{m}}\le T \right\} \ge \varepsilon\;{\mathrm{for}}\;{\mathrm{all}}\;m\ge {{m}_{1}}. \end{aligned}$$

Define a non-negative ${{C}^{2}}$ function $V:\mathbb {R}_{+}^{2}\rightarrow {{\bar{\mathbb {R}}}_{+}}$ as follows

9 $$\begin{aligned} \left( x,y \right) =\left( x-1-\ln x \right) +\left( y-1-\ln y \right) . \end{aligned}$$

Assuming that $m\ge {{m}_{1}}$ and $T>0$ be arbitrary. For any $0\le t\le \min \left\{ {{\tau }_{m}},\ T \right\}$, by using It’s formula,

10 $$\begin{aligned} dV\left( x,y \right)&:=LV\left( x,y \right) +\sigma ydB\left( t \right) -\sigma xdB\left( t \right) \\&\quad -\int _{\mathbb {Y}}{\left[ \gamma \left( u \right) xy+\ln \left( 1-\gamma \left( u \right) y \right) \right] }\tilde{N}\left( dt,du \right) \\&\quad +\int _{\mathbb {Y}}{\left[ \gamma \left( u \right) xy-\ln \left( 1+\gamma \left( u \right) x \right) \right] }\tilde{N}\left( dt,du \right) , \end{aligned}$$

where $LV:\mathbb {R}_{+}^{2}\rightarrow \mathbb {R}$ is defined by

$$\begin{aligned} LV\left( x,y \right)&=\left( 1-\frac{1}{x} \right) \left( -{{a}_{1}}x+{{b}_{1}}y+{{c}_{1}}xy \right) +\left( 1-\frac{1}{y} \right) \left( {{a}_{1}}x-{{b}_{2}}y+{{c}_{2}}xy-{{k}_{2}}{{y}^{2}} \right) \\&\quad -\int _{\mathbb {Y}}{\left[ \ln \left( 1-\gamma \left( u \right) y \right) +\gamma \left( u \right) y \right] }\lambda \left( du \right) +\frac{{{\sigma }^{2}}}{2}{{x}^{2}} \\&\quad -\int _{\mathbb {Y}}{\left[ \ln \left( 1+\gamma \left( u \right) x \right) -\gamma \left( u \right) x \right] }\lambda \left( du \right) +\frac{{{\sigma }^{2}}}{2}{{y}^{2}} \\&\le {{k}_{1}}{{a}_{0}}+{{k}_{-1}}{{x}_{0}}+{{k}_{2}}\left( {{c}_{0}}-{{x}_{0}} \right) +\left( {{k}_{-1}}+{{k}_{1}}+{{k}_{2}} \right) \left( x+y \right) +\frac{{{\sigma }^{2}}}{2}\left( {{x}^{2}}+{{y}^{2}} \right) \\&\quad -\int _{\mathbb {Y}}{\left[ \ln \left( 1-\gamma \left( u \right) y \right) +\gamma \left( u \right) y \right] }\lambda \left( du \right) \\&\quad -\int _{\mathbb {Y}}{\left[ \ln \left( 1+\gamma \left( u \right) x \right) -\gamma \left( u \right) x \right] }\lambda \left( du \right) \\&\le {{k}_{1}}{{a}_{0}}+{{k}_{-1}}{{x}_{0}}+{{k}_{2}}\left( {{c}_{0}}-{{x}_{0}} \right) +k\left( \frac{{{k}_{2}}\left( {{x}_{0}}-{{c}_{0}} \right) }{2{{k}_{-1}}-{{k}_{2}}} \right) \\&\quad +{{\sigma }^{2}}{{\left( \frac{{{k}_{2}}\left( {{x}_{0}}-{{c}_{0}} \right) }{2{{k}_{-1}}-{{k}_{2}}} \right) }^{2}}+{{H}_{1}}+{{H}_{2}}, \end{aligned}$$

in which

$$\begin{aligned} & H_{1} = - \int_{ {\mathbb{Y}}} {\left[ {\ln \left( {1 - \gamma (u)y} \right) + \gamma (u)y} \right]} \lambda (du), \\ & H_{2} = - \int_{ {\mathbb{Y}}} {\left[ {\ln \left( {1 + \gamma (u)x} \right) - \gamma (u)x} \right]} \lambda (du), \\ & k = k_{1} + k_{{ - 1}} + k_{2} . \\ \end{aligned}$$

According @@to Assumption 1, we have $1-\gamma \left( u \right) y>0$ for any $u\in \mathbb {Y}$. On the basis of Taylor’s formula and Assumption 1, we can get the following conclusion

$$\begin{aligned} {{H}_{1}}&=\int _{\mathbb {Y}}{\left[ \gamma \left( u \right) y-\gamma \left( u \right) y+\frac{{{\gamma }^{2}}\left( u \right) {{y}^{2}}}{2!{{\left( 1-\gamma \left( u \right) \left( 0+\theta \left( y-0 \right) \right) \right) }^{2}}} \right] }\lambda \left( du \right) \\&=\int _{\mathbb {Y}}{\left[ \frac{{{\gamma }^{2}}\left( u \right) {{y}^{2}}}{2{{\left( 1-\gamma \left( u \right) y\theta \right) }^{2}}} \right] }\lambda \left( du \right) \\&\le \frac{{{\delta }^{2}}}{2{{\left( 1-\delta \right) }^{2}}}\lambda \left( \mathbb {Y} \right) , \end{aligned}$$

where $\theta \in \left( 0,\ 1 \right)$ is an arbitrary number.

In the same way, it can be concluded that

$$\begin{aligned} {{H}_{2}}=\int _{\mathbb {Y}}{\left[ \frac{{{\gamma }^{2}}\left( u \right) {{x}^{2}}}{2{{\left( 1+\gamma \left( u \right) \theta x \right) }^{2}}} \right] }\lambda \left( du \right) \le \frac{{{\delta }^{2}}}{2{{\left( 1-\delta \right) }^{2}}}\lambda \left( \mathbb {Y} \right) \end{aligned}$$

Then, it is concluded that

$$\begin{aligned} LV\left( x,y \right)&\le {{k}_{1}}{{a}_{0}}+{{k}_{-1}}{{x}_{0}}+{{k}_{2}}\left( {{c}_{0}}-{{x}_{0}} \right) +k\left( \frac{{{k}_{2}}\left( {{x}_{0}}-{{c}_{0}} \right) }{2{{k}_{-1}}-{{k}_{2}}} \right) \\&\quad +{{\sigma }^{2}}{{\left( \frac{{{k}_{2}}\left( {{x}_{0}}-{{c}_{0}} \right) }{2{{k}_{-1}}-{{k}_{2}}} \right) }^{2}}+\frac{{{\delta }^{2}}}{{{\left( 1-\delta \right) }^{2}}}\lambda \left( \mathbb {Y} \right) \\&:=C \end{aligned}$$

where *C* is a constant.

11 $$\begin{aligned} \int _{0}^{{{\tau }_{m}}\wedge T}&{dV\left( x\left( t \right) ,y\left( t \right) \right) \le \int _{0}^{{{\tau }_{m}}\wedge T}{Cds}+}\int _{0}^{{{\tau }_{m}}\wedge T}{\sigma y\left( {{s}^{-}} \right) dB\left( s \right) }\\&-\int _{0}^{{{\tau }_{m}}\wedge T}{\sigma x\left( {{s}^{-}} \right) dB\left( s \right) } \\&-\int _{0}^{{{\tau }_{m}}\wedge T}{\int _{\mathbb {Y}}{\left[ \gamma \left( u \right) x\left( {{s}^{-}} \right) y\left( {{s}^{-}} \right) -\ln \left( 1-\gamma \left( u \right) y\left( {{s}^{-}} \right) \right) \right] \tilde{N}\left( ds,du \right) }} \\&+\int _{0}^{{{\tau }_{m}}\wedge T}{\int _{\mathbb {Y}}{\left[ \gamma \left( u \right) x\left( {{s}^{-}} \right) y\left( {{s}^{-}} \right) -\ln \left( 1+\gamma \left( u \right) x\left( {{s}^{-}} \right) \right) \right] \tilde{N}\left( ds,du \right) }} \\ \end{aligned}$$

Taking expectations from both sides of (11), we obtain

12 $$\begin{aligned} EV\left( x\left( {{\tau }_{m}}\wedge T \right) ,y\left( {{\tau }_{m}}\wedge T \right) \right)&\le V\left( x\left( 0 \right) ,y\left( 0 \right) \right) +CE\left( {{\tau }_{m}}\wedge T \right) \\&\le V\left( x\left( 0 \right) ,y\left( 0 \right) \right) +CT. \end{aligned}$$

Set ${{\Omega }_{m}}=\left\{ {{\tau }_{m}}\le T \right\}$ for $m\ge {{m}_{1}}$ and according to Eq. (8), we have $P\left\{ {{\Omega }_{m}} \right\} \ge \varepsilon$. Note that for every $\omega \in {{\Omega }_{m}}$, $x\left( {{\tau }_{m}},\ \omega \right)$ or $y\left( {{\tau }_{m}},\ \omega \right)$ is equal to one of *m* or $\frac{1}{m}$. So $V\left( x\left( {{\tau }_{m}},\ \omega \right) ,y\left( {{\tau }_{m}},\ \omega \right) \right)$ is not less than either

$$m-1-\ln m\;{\mathrm{or}}\;\frac{1}{m}-1-\ln \frac{1}{m}=\frac{1}{m}-1+\ln m.$$

Therefore,

13 $$\begin{aligned} V\left( x\left( {{\tau }_{m}},\omega \right) ,y\left( {{\tau }_{m}},\omega \right) \right) \ge \left( m-1-\ln m \right) \wedge \left( \frac{1}{m}-1+\ln m \right) \end{aligned}$$

It can be seen from (12),

$$\begin{aligned} V\left( x\left( 0 \right) ,y\left( 0 \right) \right) +CT&\ge E\left[ {{I}_{{{\Omega }_{m}}}}V\left( x\left( {{\tau }_{m}},\omega \right) ,y\left( {{\tau }_{m}},\omega \right) \right) \right] \\&\ge \varepsilon \left[ \left( m-1-\ln m \right) \wedge \left( \frac{1}{m}-1+\ln m \right) \right] \end{aligned}$$

where ${{I}_{{{\Omega }_{m}}}}$ indicates the indicator function of ${{\Omega }_{m}}$. Here $m\rightarrow \infty$ leads to the contradiction

$$\begin{aligned} \infty >V\left( x\left( 0 \right) ,y\left( 0 \right) \right) +CT=\infty , \end{aligned}$$

so we must have ${{\tau }_{m}}=\infty\; a.s.$ This completes the proof. $\square$

### Remark 4

According to system (6), we have

$$\begin{aligned} d\left( x+y \right) \le \left[ {{k}_{2}}\left( {{c}_{0}}-{{x}_{0}} \right) +\left( 2{{k}_{-1}}-{{k}_{2}} \right) \left( x+y \right) \right] dt. \end{aligned}$$

Therefore the region

$$\begin{aligned} {{\Gamma }^{*}}=\left\{ \left( x,y \right) \in \mathbb {R}_{+}^{2}:x+y<\frac{{{k}_{2}}\left( {{x}_{0}}-{{c}_{0}} \right) }{2{{k}_{-1}}-{{k}_{2}}} \right\} \end{aligned}$$

is a positive invariant set of system (6). In the following, set the initial value $x\left( 0 \right) +y\left( 0 \right) \in {{\Gamma }^{*}}$of system (6).

## Sufficient conditions for end and continuation of reaction

In order to study the effect of Lévy noise on EDA circuit response, the changes in the response of EDA circuit and the reactivity of ThTSignal under different noise intensities are analyzed below.

### Conditions for the end of reaction

In this section, the sufficient conditions for the end of the reaction of system (6) is proved. First of all, the following theorems are given.

#### Theorem 2

Under Assumption 1, assuming that $\left( x\left( t \right) ,y\left( t \right) \right)$ be the solution of system (6) with any given initial value $\left( x\left( 0 \right) ,y\left( 0 \right) \right) \in {{\Gamma }^{*}}$. If one of the following two conditions is true.

$$\left( a \right) {{{\sigma }^{\prime }}^{2}}>\frac{c_{1}^{2}}{2{{b}_{2}}}$$, or

$$\left( b \right) {{{\sigma }^{\prime }}^{2}}\le \frac{{{c}_{1}}}{\frac{{{k}_{2}}\left( {{x}_{0}}-{{c}_{0}} \right) }{2{{k}_{-1}}-{{k}_{2}}}}$$ and $$\frac{{{c}_{1}}}{{{b}_{2}}}\left( \frac{{{k}_{2}}\left( {{x}_{0}}-{{c}_{0}} \right) }{2{{k}_{-1}}-{{k}_{2}}} \right) -\frac{{{{{\sigma }^{\prime }}}^{2}}}{2{{b}_{2}}}{{\left( \frac{{{k}_{2}}\left( {{x}_{0}}-{{c}_{0}} \right) }{2{{k}_{-1}}-{{k}_{2}}} \right) }^{2}}<1$$, then

$$\underset{t\rightarrow \infty }{\mathop {\lim \sup }}\,\frac{\ln y\left( t \right) }{t}\le \frac{c_{1}^{2}}{2{{{{\sigma }^{\prime }}}^{2}}}+{{b}_{2}}$$ (*a*.*s*.), if $$\left( a \right)$$ holds,

$\underset{t\rightarrow \infty }{\mathop {\lim \sup }}\,\frac{\ln y\left( t \right) }{t}\le {{b}_{2}}\left[ -1+\frac{{{c}_{1}}}{{{b}_{2}}}\left( \frac{{{k}_{2}}\left( {{x}_{0}}-{{c}_{0}} \right) }{2{{k}_{-1}}-{{k}_{2}}} \right) -\frac{{{{{\sigma }^{\prime }}}^{2}}}{2{{b}_{2}}}{{\left( \frac{{{k}_{2}}\left( {{x}_{0}}-{{c}_{0}} \right) }{2{{k}_{-1}}-{{k}_{2}}} \right) }^{2}} \right] <0$ (*a*.*s*.),  if $\left( b \right)$ holds, where ${{{\sigma }^{\prime }}^{2}}={{\sigma }^{2}}+\int _{\mathbb {Y}}{\frac{{{\gamma }^{2}}\left( u \right) }{{{\left( 1+\delta \right) }^{2}}}\lambda \left( du \right) }$. This means that the reaction will end with an exponential probability of 1.

#### Proof

From model (6),

$$\begin{aligned} \frac{x\left( t \right) -x\left( 0 \right) }{t}+\frac{y\left( t \right) -y\left( 0 \right) }{t}&={{k}_{2}}\left( {{x}_{0}}-{{c}_{0}} \right) \left\langle y\left( {{t}^{-}} \right) \right\rangle -\left( 2{{k}_{-1}}-{{k}_{2}} \right) \left\langle x\left( {{t}^{-}} \right) y\left( {{t}^{-}} \right) \right\rangle \\&-{{k}_{2}}\left\langle {{y}^{2}}\left( {{t}^{-}} \right) \right\rangle \end{aligned}$$

and then

14 $$\begin{aligned} \left\langle x\left( {{t}^{-}} \right) \right\rangle =\frac{{{k}_{2}}\left( {{x}_{0}}-{{c}_{0}} \right) }{2{{k}_{-1}}-{{k}_{2}}}-\frac{{{k}_{2}}}{2{{k}_{-1}}-{{k}_{2}}}\left\langle y\left( {{t}^{-}} \right) \right\rangle +\varphi \left( t \right) , \end{aligned}$$

where

15 $$\begin{aligned} \varphi \left( t \right) =-\frac{1}{\left( 2{{k}_{-1}}-{{k}_{2}} \right) \left\langle y\left( {{t}^{-}} \right) \right\rangle }\left[ \frac{x\left( t \right) -x\left( 0 \right) }{t}+\frac{y\left( t \right) -y\left( 0 \right) }{t} \right] \end{aligned}$$

satisfies

16 $$\begin{aligned} \underset{t\rightarrow \infty }{\mathop {\lim }}\,\varphi \left( t \right) =0. \end{aligned}$$

Using It’s formula to calculate $\ln y$, we can get the following results

$$\begin{aligned} d\left( \ln y \right)&=\left\{ \frac{{{a}_{1}}x\left( {{t}^{-}} \right) }{y\left( {{t}^{-}} \right) }+{{b}_{2}}-{{c}_{1}}x\left( {{t}^{-}} \right) -{{k}_{2}}y\left( {{t}^{-}} \right) -\frac{{{\sigma }^{2}}}{2}{{x}^{2}}\left( {{t}^{-}} \right) \right. \\&\quad \left. +\int _{\mathbb {Y}}{\left[ \ln \left( 1+\gamma \left( u \right) x\left( {{t}^{-}} \right) \right) -\gamma \left( u \right) x\left( {{t}^{-}} \right) \right] \lambda \left( du \right) } \right\} dt \\&\quad +\sigma x\left( {{t}^{-}} \right) dB\left( t \right) +\int _{\mathbb {Y}}{\left[ \ln \left( 1+\gamma \left( u \right) x\left( {{t}^{-}} \right) \right) \right] \tilde{N}\left( dt,du \right) .} \end{aligned}$$

Integrate this equation from 0 to t, and divide both sides by t to get

17 $$\begin{aligned}&\frac{\ln y(t) -\ln y(0) }{t}={{a}_{1}}\left\langle \frac{x\left( {{t}^{-}} \right) }{y\left( {{t}^{-}} \right) } \right\rangle +{{b}_{2}}-{{c}_{1}}\left\langle x\left( {{t}^{-}} \right) \right\rangle -{{k}_{2}}\left\langle y\left( {{t}^{-}} \right) \right\rangle -\frac{{{\sigma }^{2}}}{2}\left\langle {{x}^{2}}\left( {{t}^{-}} \right) \right\rangle \\ &\qquad +\int _{0}^{t}\int _{\mathbb{Y}}\left[\ln \left( 1+\gamma \left( u \right) x\left( {{s}^{-}} \right) \right) -\gamma \left( u \right) x\left( {{s}^{-}} \right) \right] \lambda \left( du \right) ds+\frac{{{M}_{1}}\left( t \right) }{t}+\frac{{{M}_{2}}\left( t \right) }{t}\\ &\quad \le {{b}_{2}}-{{c}_{1}}\left\langle x\left( {{t}^{-}} \right) \right\rangle -\frac{1}{2}\left[ {{\sigma }^{2}}+\int _{\mathbb{Y}}{\frac{{{\gamma }^{2}}(u)}{{{\left( 1+\delta \right) }^{2}}}\lambda \left( du \right) } \right] \left\langle {{x}^{2}}\left( {{t}^{-}} \right) \right\rangle +\frac{{{M}_{1}}( t )}{t}+\frac{{{M}_{2}}(t)}{t}\\ &\quad \le {{b}_{2}}-{{c}_{1}}\left\langle x\left( {{t}^{-}} \right) \right\rangle -\frac{{{{{\sigma }^{{\prime}2}}}}}{2}\left\langle {{x}^{2}}\left( {{t}^{-}} \right) \right\rangle +\frac{{{M}_{1}}\left( t \right) }{t}+\frac{{{M}_{2}}\left( t \right) }{t}\\ &\quad :=f\left( z \right) +\frac{{{M}_{1}}\left( t \right) }{t}+\frac{{{M}_{2}}\left( t \right) }{t}, \end{aligned}$$

where ${{M}_{1}}\left( t \right) =\sigma \int _{0}^{t}{x\left( {{s}^{-}} \right) ds},$ ${{M}_{2}}\left( t \right) =\int _{0}^{t}{\int _{\mathbb {Y}}{\left[ \ln \left( 1+\gamma \left( u \right) x\left( {{s}^{-}} \right) \right) \right] \tilde{N}\left( ds,du \right) }}$are all martingale terms and $f:\left( 0\frac{{{k}_{2}}\left( {{x}_{0}}-{{c}_{0}} \right) }{2{{k}_{-1}}-{{k}_{2}}} \right) \rightarrow \mathbb {R}$ is defined by

18 $$\begin{aligned} f\left( z \right)&={{b}_{2}}+{{c}_{1}}z-\frac{{{{{\sigma }^{\prime }}}^{2}}}{2}{{z}^{2}}\\&=-\frac{{{{{\sigma }^{\prime }}}^{2}}}{2}{{\left( z-\frac{{{c}_{1}}}{{{{{\sigma }^{\prime }}}^{2}}} \right) }^{2}}+\frac{c_{1}^{2}}{2{{{{\sigma }^{\prime }}}^{2}}}+{{b}_{2}}z\\&=\left\langle x\left( {{t}^{-}} \right) \right\rangle \in \left( 0\frac{{{k}_{2}}\left( {{x}_{0}}-{{c}_{0}} \right) }{2{{k}_{-1}}-{{k}_{2}}} \right) . \end{aligned}$$

**Case 1** When ${{{\sigma }^{\prime }}^{2}}>\frac{c_{1}^{2}}{2{{b}_{2}}}$, by (18), one can see that

19 $$\begin{aligned} f\left( z \right) \le f\left( \frac{{{c}_{1}}}{{{{{\sigma }^{\prime }}}^{2}}} \right) =\frac{c_{1}^{2}}{2{{{{\sigma }^{\prime }}}^{2}}}+{{b}_{2}}, \end{aligned}$$

Then from (17), we have

20 $$\begin{aligned} \frac{\ln y\left( t \right) }{t}&\le \frac{\ln y\left( 0 \right) }{t}+f\left( z \right) +\frac{{{M}_{1}}\left( t \right) }{t}+\frac{{{M}_{2}}\left( t \right) }{t} \\&\le \frac{\ln y\left( 0 \right) }{t}+\frac{c_{1}^{2}}{2{{{{\sigma }^{\prime }}}^{2}}}+{{b}_{2}}+\frac{{{M}_{1}}\left( t \right) }{t}+\frac{{{M}_{2}}\left( t \right) }{t}. \end{aligned}$$

The quadratic variation of ${{M}_{i}}\left( t \right) ,\ i=1,2$can be calculated

$$\begin{aligned} {{\left\langle {{M}_{1}},{{M}_{1}} \right\rangle }_{t}}&={{\sigma }^{2}}\int _{0}^{t}{{{x}^{2}}\left( {{s}^{-}} \right) ds\le }{{\sigma }^{2}}{{\left( \frac{{{k}_{2}}\left( {{x}_{0}}-{{c}_{0}} \right) }{2{{k}_{-1}}-{{k}_{2}}} \right) }^{2}}t,\\ {{\left\langle {{M}_{2}},{{M}_{2}} \right\rangle }_{t}}&=\int _{0}^{t}{\int _{\mathbb {Y}}{\left[ \ln {{\left( 1+\gamma \left( u \right) x\left( {{s}^{-}} \right) \right) }^{2}} \right] }\lambda \left( du \right) ds} \\&\le \max \left\{ \left[ \ln {{\left( 1-\delta \right) }^{2}} \right] ,\left[ \ln {{\left( 1+\delta \right) }^{2}} \right] \right\} \lambda \left( \mathbb {Y} \right) t. \end{aligned}$$

According to the strong law of large numbers of local martingale, it follows that

21 $$\begin{aligned} \underset{t\rightarrow \infty }{\mathop {\lim }}\,\frac{{{M}_{i}}\left( t \right) }{t}=0\;a.s.\; i=1,2. \end{aligned}$$

Taking the upper limit on both sides of Eq. (20), we get

22 $$\begin{aligned} \underset{t\rightarrow \infty }{\mathop {\lim \sup }}\,\frac{\ln y\left( t \right) }{t}\le \frac{c_{1}^{2}}{2{{{{\sigma }^{\prime }}}^{2}}}+{{b}_{2}}<0\;a.s. \end{aligned}$$

**Case 2** when ${{{\sigma }^{\prime }}^{2}}\le \frac{{{c}_{1}}}{\frac{{{k}_{2}}\left( {{x}_{0}}-{{c}_{0}} \right) }{2{{k}_{-1}}-{{k}_{2}}}}$ and $\frac{{{c}_{1}}}{{{b}_{2}}}\left( \frac{{{k}_{2}}\left( {{x}_{0}}-{{c}_{0}} \right) }{2{{k}_{-1}}-{{k}_{2}}} \right) -\frac{{{{{\sigma }^{\prime }}}^{2}}}{2{{b}_{2}}}{{\left( \frac{{{k}_{2}}\left( {{x}_{0}}-{{c}_{0}} \right) }{2{{k}_{-1}}-{{k}_{2}}} \right) }^{2}}<1$, from equation (18), it’s easy to see that

$$\begin{aligned} f\left( z \right)&\le f\left( \frac{{{k}_{2}}\left( {{x}_{0}}-{{c}_{0}} \right) }{2{{k}_{-1}}-{{k}_{2}}} \right) =-{{b}_{2}}+{{c}_{1}}\left( \frac{{{k}_{2}}\left( {{x}_{0}}-{{c}_{0}} \right) }{2{{k}_{-1}}-{{k}_{2}}} \right) -\frac{{{{{\sigma }^{\prime }}}^{2}}}{2}{{\left( \frac{{{k}_{2}}\left( {{x}_{0}}-{{c}_{0}} \right) }{2{{k}_{-1}}-{{k}_{2}}} \right) }^{2}}\\&={{b}_{2}}\left[ -1+\frac{{{c}_{1}}}{{{b}_{2}}}\left( \frac{{{k}_{2}}\left( {{x}_{0}}-{{c}_{0}} \right) }{2{{k}_{-1}}-{{k}_{2}}} \right) -\frac{{{{{\sigma }^{\prime }}}^{2}}}{2{{b}_{2}}}{{\left( \frac{{{k}_{2}}\left( {{x}_{0}}-{{c}_{0}} \right) }{2{{k}_{-1}}-{{k}_{2}}} \right) }^{2}} \right] . \end{aligned}$$

Similarly, $$ {\text{lim}}_{{t \to \infty }}\sup\,\frac{\ln y\left( t \right) }{t}\le {{b}_{2}}\left[ -1+\frac{{{c}_{1}}}{{{b}_{2}}}\left( \frac{{{k}_{2}}\left( {{x}_{0}}-{{c}_{0}} \right) }{2{{k}_{-1}}-{{k}_{2}}} \right) -\frac{{{{{\sigma }^{\prime }}}^{2}}}{2{{b}_{2}}}{{\left( \frac{{{k}_{2}}\left( {{x}_{0}}-{{c}_{0}} \right) }{2{{k}_{-1}}-{{k}_{2}}} \right) }^{2}} \right] <0\;a.s.$$

In summary, ${\text{lim}}_{{t \to \infty }} y\left( t \right) = 0\; a.s.$ This completes the proof. $\square$

#### Remark 5

It can be known from Theorem 3 that if the intensity of the noise is large enough so that ${{{\sigma }^{\prime }}^{2}}>\frac{c_{1}^{2}}{2{{b}_{2}}}$ or the intensity of the noise meets conditions (b), EDA circuit reaction will end in exponential form with probability 1. This shows that when the above conditions are met, Lévy jump will force EDA circuit to end the reaction.

#### Remark 6

When EDA circuit is completed, the reactants of biological information system are consumed. At this time, the resulting product concentration reaches the maximum, and the system’s unbaled fluorescence reaction is the strongest, indicating that the reaction activity of unlabeled fluorescent reporter ThTSignal reaches the maximum.

### Continuous reaction conditions

In this section, the conditions under which system (6) reaction continues are considered. First, give the following definition

#### Definition 1

System (6) is persistent if $\underset{t\rightarrow \infty }{\mathop {\lim \inf }}\,\int _{0}^{t}{y\left( s \right) ds}>0\;a.s.$

#### Assumption 2

$$R_{0}^{*}:={{R}_{0}}-\frac{{{{{\sigma }''}}^{2}}}{2{{b}_{2}}}{{\left( \frac{{{k}_{2}}\left( {{x}_{0}}-{{c}_{0}} \right) }{2{{k}_{-1}}-{{k}_{2}}} \right) }^{2}}>1,\;{\mathrm{where}}\;{{{\sigma }''}^{2}}={{\sigma }^{2}}+\int _{\mathbb {Y}}{\frac{{{\gamma }^{2}}\left( u \right) }{{{\left( 1-\delta \right) }^{2}}}\lambda \left( du \right) .}$$

#### Theorem 3

Let Assumptions 1 and 2 hold, $\left( x\left( 0\right) ,y\left( 0\right) \right) \in {{\Gamma }^{*}}$ is any given initial value, the solution $\left( x\left( t \right) ,y\left( t \right) \right)$ of system (6) has the following property

$$\underset{t\rightarrow \infty }{\mathop {\lim \inf }}\,\left\langle y\left( {{t}^{-}} \right) \right\rangle \ge \frac{2{{k}_{-1}}+{{k}_{2}}}{{{k}_{2}}}\left[ \frac{{{b}_{2}}}{{{c}_{1}}}-\frac{{{{{\sigma }''}}^{2}}}{2{{c}_{1}}}{{\left( \frac{{{k}_{2}}\left( {{x}_{0}}-{{c}_{0}} \right) }{2{{k}_{-1}}-{{k}_{2}}} \right) }^{2}} \right] +\left( {{x}_{0}}-{{c}_{0}} \right) >0.$$

#### Proof

In term of the first equation of (17), we can get

23 $$\begin{aligned} \frac{\ln y\left( t \right) -\ln y\left( 0 \right) }{t}&={{a}_{1}}\left\langle \frac{x\left( {{t}^{-}} \right) }{y\left( {{t}^{-}} \right) } \right\rangle +{{b}_{2}}-{{c}_{1}}\left\langle x\left( {{t}^{-}} \right) \right\rangle -{{k}_{2}}\left\langle y\left( {{t}^{-}} \right) \right\rangle \\&\quad -\frac{{{\sigma }^{2}}}{2}\left\langle {{x}^{2}}\left( {{t}^{-}} \right) \right\rangle +\frac{{{M}_{1}}\left( t \right) }{t}+\frac{{{M}_{2}}\left( t \right) }{t} \\&\quad +\frac{1}{t}\int _{0}^{t}{\int _{\mathbb {Y}}{\left[ \ln \left( 1+\gamma \left( u \right) x\left( {{s}^{-}} \right) \right) -\gamma \left( u \right) x\left( {{s}^{-}} \right) \right] \lambda \left( du \right) }}ds \\&\ge {{b}_{2}}-{{c}_{1}}\left\langle x\left( {{t}^{-}} \right) \right\rangle -\frac{{{\sigma }^{2}}}{2}{{\left( \frac{{{k}_{2}}\left( {{x}_{0}}-{{c}_{0}} \right) }{2{{k}_{-1}}-{{k}_{2}}} \right) }^{2}}\\&\quad -{{\left( \frac{{{k}_{2}}\left( {{x}_{0}}-{{c}_{0}} \right) }{2{{k}_{-1}}-{{k}_{2}}} \right) }^{2}}\int _{\mathbb {Y}}{\frac{{{\gamma }^{2}}\left( u \right) }{2{{\left( 1-\delta \right) }^{2}}}\lambda \left( du \right) }+\frac{{{M}_{1}}\left( t \right) }{t}+\frac{{{M}_{2}}\left( t \right) }{t} \\&={{b}_{2}}-{{c}_{1}}\left\langle x\left( {{t}^{-}} \right) \right\rangle -\frac{1}{2}\left( {{\sigma }^{2}}+\int _{\mathbb {Y}}{\frac{{{\gamma }^{2}}\left( u \right) }{{{\left( 1-\delta \right) }^{2}}}\lambda \left( du \right) } \right) {{\left( \frac{{{k}_{2}}\left( {{x}_{0}}-{{c}_{0}} \right) }{2{{k}_{-1}}-{{k}_{2}}} \right) }^{2}}\\&\quad +\frac{{{M}_{1}}\left( t \right) }{t}+\frac{{{M}_{2}}\left( t \right) }{t} \\&={{b}_{2}}-{{c}_{1}}\left\langle x\left( {{t}^{-}} \right) \right\rangle -\frac{{{{{\sigma }''}}^{2}}}{2}{{\left( \frac{{{k}_{2}}\left( {{x}_{0}}-{{c}_{0}} \right) }{2{{k}_{-1}}-{{k}_{2}}} \right) }^{2}}+\frac{{{M}_{1}}\left( t \right) }{t}+\frac{{{M}_{2}}\left( t \right) }{t}. \end{aligned}$$

Substitute (14) into (23) to get

$$\begin{aligned} \frac{\ln y\left( t \right) -\ln y\left( 0 \right) }{t}&\ge {{b}_{2}}-{{c}_{1}}\left[ \frac{{{k}_{2}}\left( {{x}_{0}}-{{c}_{0}} \right) }{2{{k}_{-1}}-{{k}_{2}}}-\frac{{{k}_{2}}}{2{{k}_{-1}}-{{k}_{2}}}\left\langle y\left( {{t}^{-}} \right) \right\rangle +\varphi \left( t \right) \right] \\&\quad -\frac{{{{{\sigma }''}}^{2}}}{2}{{\left( \frac{{{k}_{2}}\left( {{x}_{0}}-{{c}_{0}} \right) }{2{{k}_{-1}}-{{k}_{2}}} \right) }^{2}}+\frac{{{M}_{1}}\left( t \right) }{t}+\frac{{{M}_{2}}\left( t \right) }{t}. \end{aligned}$$

Since $-\infty<\ln y\left( t \right) <\ln \left( \frac{{{k}_{2}}\left( {{x}_{0}}-{{c}_{0}} \right) }{2{{k}_{-1}}-{{k}_{2}}} \right)$, the above formula can be rewritten as

24 $$\begin{aligned} \left\langle y\left( {{t}^{-}} \right) \right\rangle&\ge \frac{2{{k}_{-1}}+{{k}_{2}}}{{{k}_{2}}}\left\{ \frac{\ln y\left( t \right) -\ln y\left( 0 \right) }{{{c}_{1}}t}-\frac{{{b}_{2}}}{{{c}_{1}}}+\frac{{{{{\sigma }''}}^{2}}}{2{{c}_{1}}}{{\left( \frac{{{k}_{2}}\left( {{x}_{0}}-{{c}_{0}} \right) }{2{{k}_{-1}}-{{k}_{2}}} \right) }^{2}} \right. \\&\quad \left. -\frac{{{M}_{1}}\left( t \right) }{{{c}_{1}}t}-\frac{{{M}_{2}}\left( t \right) }{{{c}_{1}}t}-\frac{{{k}_{2}}\left( {{x}_{0}}-{{c}_{0}} \right) }{2{{k}_{-1}}-{{k}_{2}}}-\varphi \left( t \right) \right\} . \end{aligned}$$

Take the lower limit on both sides of the above formula to get $\underset{t\rightarrow \infty }{\mathop {\lim \inf }}\,\left\langle y\left( {{t}^{-}} \right) \right\rangle \ge \frac{2{{k}_{-1}}-{{k}_{2}}}{{{k}_{2}}}\left[ \frac{{{b}_{2}}}{{{c}_{1}}}-\frac{{{{{\sigma }''}}^{2}}}{2{{c}_{1}}}{{\left( \frac{{{k}_{2}}\left( {{x}_{0}}-{{c}_{0}} \right) }{2{{k}_{-1}}-{{k}_{2}}} \right) }^{2}} \right] +\left( {{x}_{0}}-{{c}_{0}} \right) .$

In light of the condition $R_{0}^{*}>1$, $\underset{t\rightarrow \infty }{\mathop {\lim \inf }}\,\left\langle y\left( {{t}^{-}} \right) \right\rangle >0$ can be obtained. This completes the proof. $\square$

#### Remark 7

According to Theorem 3, as long as the noise is small enough to satisfy $R_{0}^{*}={{R}_{0}}-\frac{{{{{\sigma }''}}^{2}}}{2{{b}_{2}}}{{\left( \frac{{{k}_{2}}\left( {{x}_{0}}-{{c}_{0}} \right) }{2{{k}_{-1}}-{{k}_{2}}} \right) }^{2}}>1$, EDA circuit reaction will continue. The concentration of reactants in EDA circuit reaches a dynamic equilibrium, which indicates that the reactant remains. At this point, the reaction activity of unlabeled fluorescent reporter ThTSignal is low, and the fluorescence in-tensity of EDA circuit reaction is weak. The persistence of the reaction will not be conducive to the monitoring of EDA circuit reaction.

#### Remark 8

When the conditions of Theorem 2 are satisfied, Lévy noise will force the reaction to end earlier. At this point, the reaction activity of ThTSignal reaches the maximum value and the fluorescence intensity of the reaction reaches the maximum, indicating that the response has fully reacted, that is, the reaction is over. When the conditions in Theorem 3 are contented, EDA circuit reaction will continue. At this time, the reaction activity of unlabeled fluorescent reporter ThTSignal is low and the fluorescence intensity of the reaction is weak, which indicates that the reaction is not complete, but dynamic equilibrium is achieved, and EDA circuit response will continue.

#### Remark 9

In biological information system, the intensity of noise is closely related to the state of the system. Based on EDA circuit reaction, nonlinear biochemical reaction system (6) with Lévy beating is established, and sufficient conditions for the end and continuation of EDA reaction are analyzed. The results show that the end and duration of EDA circuit reaction is closely related to the intensity of Lévy noise, and Lévy jump has a significant impact on the properties of EDA circuit reaction system. When the noise intensity is large enough to make ${{{\sigma }^{\prime }}^{2}}>\frac{c_{1}^{2}}{2{{b}_{2}}}$ or noise meets conditions (b), EDA circuit response will end exponentially with probability 1, that is to force EDA circuit reaction to end earlier. When the noise is small enough to satisfy $R_{0}^{*}={{R}_{0}}-\frac{{{{{\sigma }''}}^{2}}}{2{{b}_{2}}}{{\left( \frac{{{k}_{2}}\left( {{x}_{0}}-{{c}_{0}} \right) }{2{{k}_{-1}}-{{k}_{2}}} \right) }^{2}}>1$, EDA circuit reaction will continue.

## Results

### Simulated data

In order to display EDA circuit reaction more intuitively, the following uses MATLAB software to perform a numerical simulation of system (6). Therefore, the numerical simulation of Lévy jump of system (6) is given below. Assuming the unit of time is minutes, the unit of reactant concentration is mol/L, take the initial value $\left( x\left( 0 \right) ,y\left( 0 \right) \right) =\left( 1.5\times 10^{4},\,0 \right)$, and other parameters are as follows

$${{a}_{0}}=2.8\times {{10}^{-4}}$$, $${{c}_{0}}=1.4\times {{10}^{-4}}$$, $${{k}_{1}}=2\times {{10}^{4}}$$, $${{k}_{-1}}=1.8\times {{10}^{4}}$$, $${{k}_{2}}=2.1\times {{10}^{4}}$$, $$\mathbb {Y}=\left( 0,+\infty \right)$$, $$\lambda \left( \mathbb {Y} \right) =1$$.

### Simulation study

**Case 1** Choose white noise intensity $\sigma =0.8$ and jumping noise intensity $\gamma \left( u \right) =0.55$, and set $\delta =0.7$. Meets Assumption 1 and ${{{\sigma }^{\prime }}^{2}}=5.8482>0.625=\frac{c_{1}^{2}}{2{{b}_{2}}}.$

**Fig. 2:**
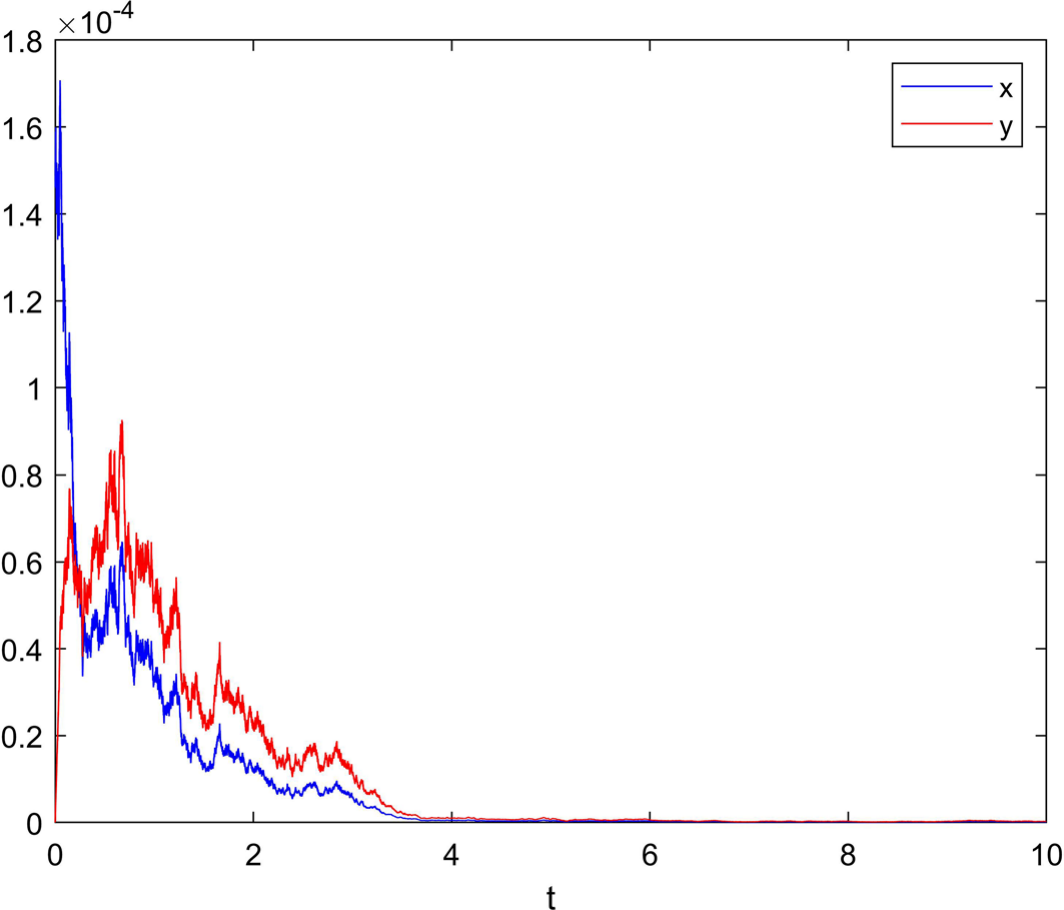
When $\sigma =0.8$ and $\gamma \left( u \right) =0.55$, the state variable response diagram of system (6)

Therefore, condition (a) in Theorem 2 is satisfied, and the reaction ends with a probability 1 index. The simulation result is shown in Fig. 2.

**Case 2** Choose white noise intensity $\sigma =0.6$, jumping noise intensity $\gamma \left( u \right) =0.75$, and set $\delta =0.1$. Meets Assumption 1 and ${{{\sigma }^{\prime }}^{2}}=0.6804\le 2.8571=\frac{{{c}_{1}}}{\frac{{{k}_{2}}\left( {{x}_{0}}-{{c}_{0}} \right) }{2{{k}_{-1}}-{{k}_{2}}}}$ and $\frac{{{c}_{1}}}{{{b}_{2}}}\left( \frac{{{k}_{2}}\left( {{x}_{0}}-{{c}_{0}} \right) }{2{{k}_{-1}}-{{k}_{2}}} \right) -\frac{{{{{\sigma }^{\prime }}}^{2}}}{2{{b}_{2}}}{{\left( \frac{{{k}_{2}}\left( {{x}_{0}}-{{c}_{0}} \right) }{2{{k}_{-1}}-{{k}_{2}}} \right) }^{2}}=0.9983<1.$

**Fig. 3:**
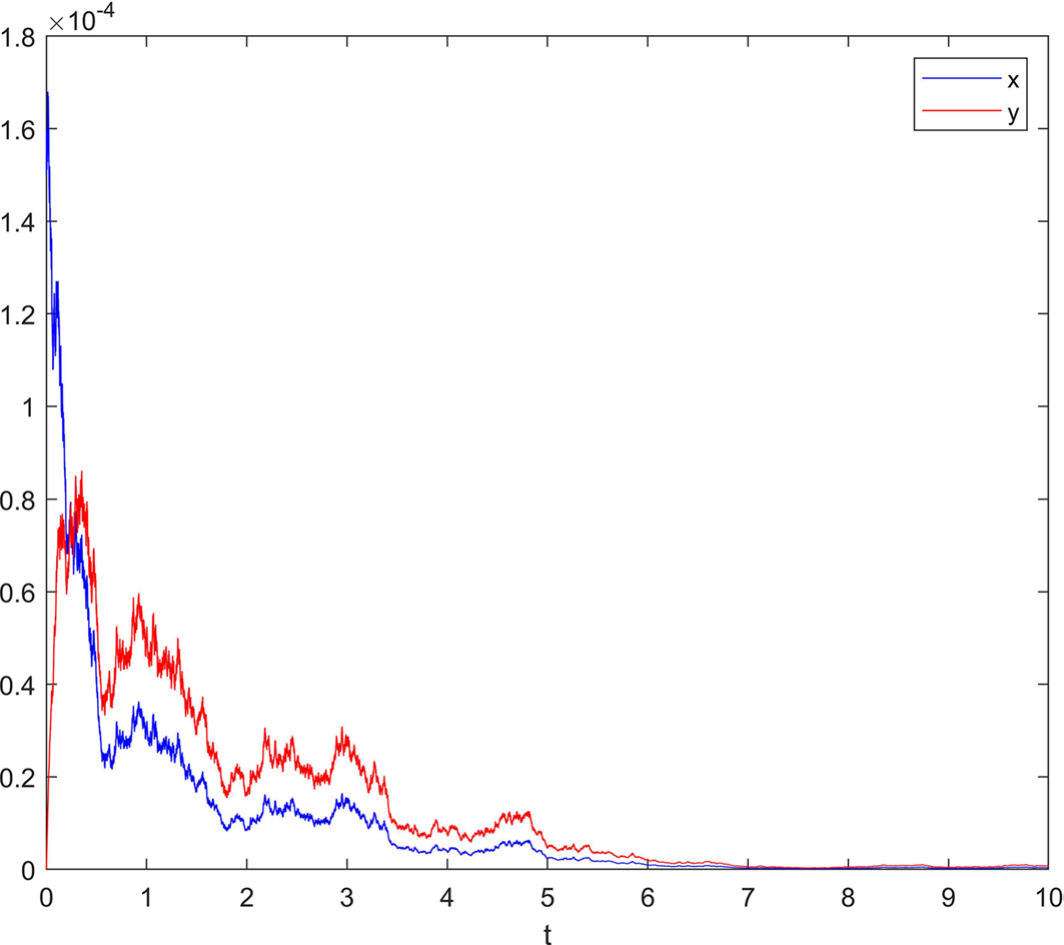
When $\sigma =0.6$ and $\gamma \left( u \right) =0.75$, the state variable response diagram of system (6)

Therefore, condition (b) in Theorem 2 is satisfied, and the reaction ends with a probability 1 index. The simulation result is shown in Fig. 3.

**Case 3** Choose white noise intensity $\sigma =0.2$, jumping noise intensity $\gamma \left( u \right) =0.15$, and set $\delta =0.2$. Meets Assumption 1 and ${{R}_{0}}-\frac{{{{{{\sigma }^{\prime }}'}}^{2}}}{2{{b}_{2}}}{{\left( \frac{{{k}_{2}}\left( {{x}_{0}}-{{c}_{0}} \right) }{2{{k}_{-1}}-{{k}_{2}}} \right) }^{2}}=1.1553>1.$

**Fig. 4:**
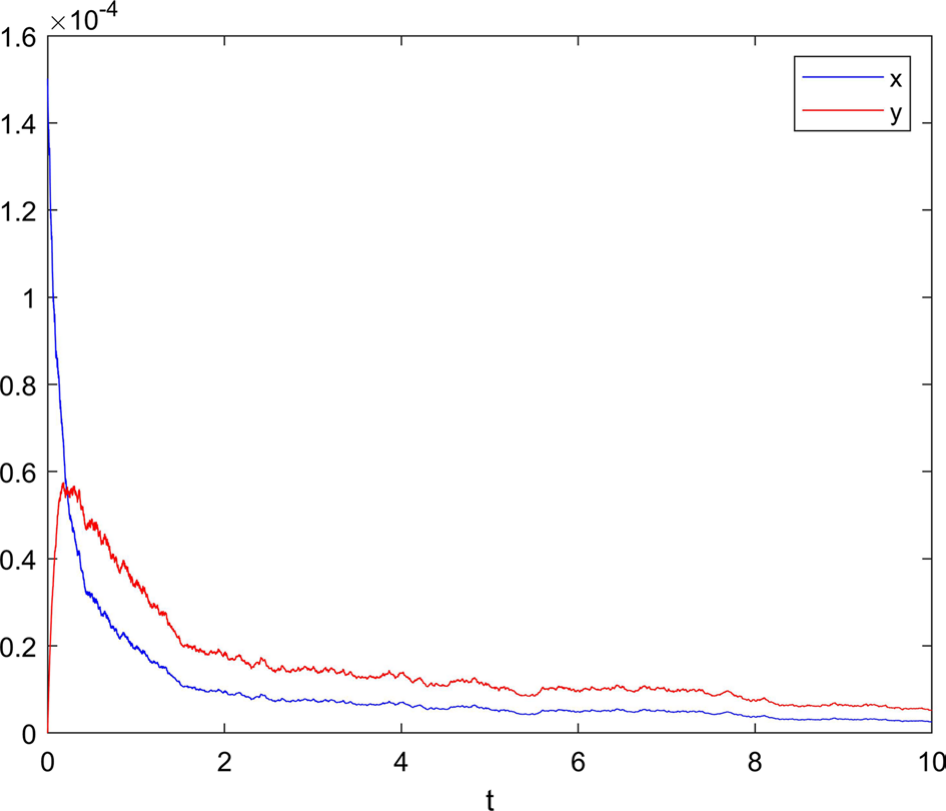
When $\sigma =0.2$ and $\gamma \left( u \right) =0.15$, the state variable response diagram of system (6)

Therefore, when EDA circuit response satisfies the condition of Theorem 3, EDA circuit response will continue. That is to say, when Lévy jump satisfies certain conditions, EDA circuit reaction will be in a dynamic equilibrium process, and with the increase of time, the concentration of the reactant will be in an equilibrium stage, that is, the concentration change will be stable, as shown in Fig. 4 of the simulation results.

#### Remark 10

When the noise intensity meets certain conditions, EDA circuit reaction will enter a state of equilibrium, at which time the system response will continue. The duration of EDA circuit response is closely related to the intensity of Lévy noise, and Lévy jump has a significant impact on biological information system.

## Discussion

From Case 1 and Case 2, it can be seen that Lévy jump will force EDA circuit to end with probability 1 when EDA circuitry meets two conditions satisfying Theorem 2. That is, EDA circuit responded in advance in advance under the influence of Lévy jump. According to Figure 2 and Figure 3, as time increases, the concentration of the reactants will also decrease until it reaches 0. At this time, the reaction of EDA circuitry will be completely consumed, so that the product concentration of EDA circuit will reach the maximum value, at which point the fluorescence intensity reaches the maximum, and the reaction activity of Thtsignal reaches the maximum. This proves that THT can be used as a non-marking reporter to complete the expression in the influence of the appropriate Lévy jump, providing a new idea for biological information.

From Case 3, when Lévy jump satisfies certain conditions, EDA circuit reaction will be in a dynamic equilibrium process, and with the increase of time, the concentration of the reactant will be in an equilibrium stage, that is, the concentration change will be stable. This shows that there are still some reactants in EDA circuit at this time, the fluorescence intensity is weak, and the reaction activity of ThTSignal is reduced.

Through the study of EDA circuit response, we can find that ThT, as an unlabeled reporter, can also promote the research of bioinformatics. Using ThT instead of fluorescent labeling can not only reduce the cost, but also regulate the fluorescence intensity of the reaction by controlling external noise, such as Lévy jump, so that we can have a more intuitive feeling. This has played a role in promoting the development of bioinformatics.

## Conclusions

In this paper, nonlinear biochemical reaction system with Lévy jump based on EDA circuit response is studied. Firstly, nonlinear biochemical reaction system model is established based on EDA circuit reaction. Considering that biochemical reaction will suffer sudden disturbances, such as sudden addition of catalyst, thermal shock and so on, in order to describe the system more accurately, nonlinear biochemical reaction system model with Lévy jump based on EDA circuit reaction is established. Then, the existence and uniqueness of positive solutions for the system is analyzed. Next, we analyze the sufficient conditions for the end of EDA circuit reaction and the sufficient conditions for the reaction to continue under the influence of Lévy jump. Finally, the conclusion is verified by numerical simulation. The results show that the end and duration of EDA circuit reaction is closely related to the intensity of Lévy noise.

## Data Availability

Not applicable.

## Abbreviations

EDA: Entropy-driven amplifier
THT: Thioflavin T

## References

[1] Pliakos K, Vens C (2019). Network inference with ensembles of bi-clustering trees. BMC Bioinform.

[2] Bai S, Du T, Khosravi E (2010). Applying internal coordinate mechanics to model the interactions between 8r-lipoxygenase and its substrate. BMC Bioinform.

[3] Dalton LA (2018). Heuristic algorithms for feature selection under Bayesian models with block-diagonal covariance structure. BMC Bioinform.

[4] Shen Y, Gong J, Li S, Liu C, Zhou L, Sheng J, Qingxia X (2021). Enzyme-free dual-DNA walker based on catalytic hairpin assembled DNAzyme for sensing telomerase activity. Sens Actuators B Chem.

[5] Ranallo S, Prévost-Tremblay C, Idili A, Vallée-Bélisle A, Ricci F (2017). Antibody-powered nucleic acid release using a DNA-based nanomachine. Nat Commun.

[6] Elbaz J, Lioubashevski O, Wang F, Remacle F, Levine RD, Willner I (2010). DNA computing circuits using libraries of DNAzyme subunits. Nat Nanotechnol.

[7] Srinivas N, Ouldridge TE, Šulc P, Schaeffer JM, Yurke B, Louis AA, Doye JP, Winfree E (2013). On the biophysics and kinetics of toehold-mediated DNA strand displacement. Nucleic Acids Res.

[8] Qian L, Winfree E, Bruck J (2011). Neural network computation with DNA strand displacement cascades. Nature.

[9] Zhang DY, Winfree E (2010). Robustness and modularity properties of a non-covalent DNA catalytic reaction. Nucleic Acids Res.

[10] Zhang DY, Winfree E (2009). Control of DNA strand displacement kinetics using toehold exchange. J Am Chem Soc.

[11] Kishi JY, Schaus TE, Gopalkrishnan N, Xuan F, Yin P (2018). Programmable autonomous synthesis of single-stranded DNA. Nat Chem.

[12] Zhang DY, Turberfield AJ, Yurke B, Winfree E (2007). Engineering entropy-driven reactions and networks catalyzed by DNA. Science.

[13] Zhang X, Zhang Q, Liu Y, Wei X (2020). A DNAzyme-mediated logic gate system based on Ag (i)-cysteine. Analyst.

[14] Wang F, Lv H, Li Q, Li J, Zhang X, Shi J, Wang L, Fan C (2020). Implementing digital computing with DNA-based switching circuits. Nat Commun.

[15] Cao B, Zhang X, Wu J, Wang B, Zhang Q, Wei X (2021). Minimum free energy coding for DNA storage. IEEE Trans Nanobiosci.

[16] Cao B, Li X, Zhang X, Wang B, Zhang Q, Wei X (2020). Designing uncorrelated address constrain for DNA storage by DMVO algorithm. IEEE/ACM Trans Comput Biol Bioinform.

[17] Capaldi S, Getts RC, Jayasena SD (2000). Signal amplification through nucleotide extension and excision on a dendritic DNA platform. Nucleic Acids Res.

[18] Wickham SF, Bath J, Katsuda Y, Endo M, Hidaka K, Sugiyama H, Turberfield AJ (2012). A DNA-based molecular motor that can navigate a network of tracks. Nat Nanotechnol.

[19] Zhang C, Wang Z, Liu Y, Yang J, Zhang X, Li Y, Pan L, Ke Y, Yan H (2019). Nicking-assisted reactant recycle to implement entropy-driven DNA circuit. J Am Chem Soc.

[20] He L, Lu D, Liang H, Xie S, Zhang X, Liu Q, Yuan Q, Tan W (2018). mRNA-initiated, three-dimensional DNA amplifier able to function inside living cells. J Am Chem Soc.

[21] Meng H-M, Shi X, Chen J, Gao Y, Qu L, Zhang K, Zhang X-B, Li Z (2020). DNA amplifier-functionalized metal–organic frameworks for multiplexed detection and imaging of intracellular mRNA. ACS Sens.

[22] Damase TR, Islam MM, Shipley M, Allen PB (2020). Thioflavin T as a noncovalent reporter for a label-free, non-enzymatic, catalytic DNA amplifier. Methods Appl Fluoresc.

[23] Hahl SK, Kremling A (2016). A comparison of deterministic and stochastic modeling approaches for biochemical reaction systems: on fixed points, means, and modes. Front Genet.

[24] Goutsias J (2007). Classical versus stochastic kinetics modeling of biochemical reaction systems. Biophys J.

[25] Kerr R, Thomson W, Smith D (2019). Mathematical modelling of the vitamin c clock reaction. R Soc Open Sci.

[26] Varfolomeev SD, Bykov VI, Semenova NA, Tsybenova SB (2020). Kinetic modeling of the blood oxygenation level dependent (bold) signals and biocatalytic reactions observed in the human brain using MRI: an analysis of normal and pathological conditions. ACS Chem Neurosci.

[27] Matlock K, De Niz C, Rahman R, Ghosh S, Pal R (2018). Investigation of model stacking for drug sensitivity prediction. BMC Bioinform.

[28] Clayton EA, Pujol TA, McDonald JF, Qiu P (2020). Leveraging TCGA gene expression data to build predictive models for cancer drug response. BMC Bioinform.

[29] Liu Y, Lv H, Wang B, Yang D, Zhang Q (2020). Modelling and analysis of haemoglobin catalytic reaction kinetic system. Math Comput Model Dyn Syst.

[30] Sivasamy P, Ganapathy JRP, Thinakaran I, Lakshmanan R (2016). Enzyme kinetic modelling and analytical solution of nonlinear rate equation in the transformation of d-methionine into l-methionine in batch reactor using the new homotopy perturbation method. Quim Nova.

[31] Bashkirtseva I, Ryashko L, Zaitseva S (2020). Stochastic sensitivity analysis of noise-induced transitions in a biochemical model with birhythmicity. J Phys A Math Theor.

[32] Elsheikh A, Wiechert W (2018). The structural index of sensitivity equation systems. Math Comput Model Dyn Syst.

[33] Dubey VP, Kumar R, Kumar D (2019). Approximate analytical solution of fractional order biochemical reaction model and its stability analysis. Int J Biomath.

[34] Nikolaev EV, Rahi SJ, Sontag ED (2018). Subharmonics and chaos in simple periodically forced biomolecular models. Biophys J.

[35] Ciesielski A, Grzywacz R (2019). Nonlinear analysis of cybernetic model for aerobic growth of saccharomyces cerevisiae in a continuous stirred tank bioreactor. Static bifurcations. Biochem Eng J.

[36] Atabaigi A, Barati A, Norouzi H (2018). Bifurcation analysis of an enzyme-catalyzed reaction–diffusion system. Comput Math Appl.

[37] Lim J, Lee S, Kim Y. Hopf bifurcation in a model of TGF-$$\beta$$ in regulation of the TH 17 phenotype. Discrete Contin Dyn Syst B. 2016;21(10):3575.

[38] Dhruba SR, Rahman A, Rahman R, Ghosh S, Pal R (2019). Recursive model for dose-time responses in pharmacological studies. BMC Bioinform.

[39] Berrhazi B-E, El Fatini M, Caraballo Garrido T, Pettersson R (2018). A stochastic SIRI epidemic model with Lévy noise. Discrete Contin Dyn Syst Ser B.

[40] Liu Y, Zhang Y, Wang Q (2020). A stochastic sir epidemic model with Lévy jump and media coverage. Adv Differ Equ.

[41] Zhao D, Yuan S (2019). Threshold dynamics of the stochastic epidemic model with jump-diffusion infection force. J Appl Anal Comput.

[42] Caraballo T, Settati A, El Fatini M, Lahrouz A, Imlahi A (2019). Global stability and positive recurrence of a stochastic sis model with Lévy noise perturbation. Physica A.

[43] Fan K, Zhang Y, Gao S, Chen S (2020). A delayed vaccinated epidemic model with nonlinear incidence rate and Lévy jumps. Physica A.

[44] Cheng Y, Li M, Zhang F (2019). A dynamics stochastic model with HIV infection of CD4+ T-cells driven by Lévy noise. Chaos Solitons Fractals.

[45] Liu C, Liu M (2019). Stochastic dynamics in a nonautonomous prey–predator system with impulsive perturbations and Lévy jumps. Commun Nonlinear Sci Numer Simul.

[46] Ma T, Meng X, Chang Z (2019). Dynamics and optimal harvesting control for a stochastic one-predator-two-prey time delay system with jumps. Complexity.

[47] Lu C, Ding X (2019). Dynamical behavior of stochastic delay Lotka-Volterra competitive model with general Lévy jumps. Physica A.

[48] Deng M (2019). Stability of a stochastic delay commensalism model with Lévy jumps. Physica A.

[49] Gao M, Jiang D (2019). Analysis of stochastic multimolecular biochemical reaction model with Lévy jumps. Physica A.

